# Is Surgical Treatment for Obstructive Sleep Apnea in Infants and Toddlers Safe? A Retrospective Comparative Analysis

**DOI:** 10.1111/coa.70087

**Published:** 2026-01-14

**Authors:** Daniel Levi, Daniel Yafit, Aviad Sapir, Yotam Heilig, Tomer Kerman, Oriya Damri, Inbal Golan‐Tripto, Daniel Michael Kaplan, Oren Ziv

**Affiliations:** ^1^ Department of Otolaryngology‐Head & Neck Surgery, Soroka University Medical Center, Faculty of Health Sciences Ben‐Gurion University of the Negev Beer Sheva Israel; ^2^ Clinical Research Center, Soroka University Medical Center and Faculty of Health Sciences Ben‐Gurion University of the Negev Beer Sheva Israel; ^3^ Pediatric Pulmonary Unit, Saban Children's Hospital, Soroka University Medical Center, Faculty of Health Sciences Ben‐Gurion University of the Negev Beer Sheva Israel

**Keywords:** adenoidectomy, complication, infants, OSA, toddlers, tonsillectomy

## Abstract

**Objective:**

To assess if surgery for Obstructive Sleep Apnea Disorder (OSAD) is safe for infants and toddlers.

**Methods:**

Retrospective cohort study of paediatric patients undergoing OSA surgery; partial or complete tonsillectomy with adenoidectomy, tonsillectomy without adenoidectomy and adenoidectomy. Patients were divided into three groups: infants (≤ 1 year), toddlers (1–2 years) and control (children ≥ 2 years). The study measured outcomes such as paediatric intensive care unit (PICU) admissions, length of hospital stay (LOS), emergency room (ER) visits within 2 weeks of surgery, fever, dehydration, bleeding and the need for reoperation.

**Results:**

A total of 419 paediatric patients were included: 61 infants (14.5%), 147 toddlers (35.1%) and 211 controls (50.4%). Adenoidectomy was the most common procedure for infants (75%), followed by toddlers (52%) and controls (27%). The LOS was significantly longer in the infant group (*p* < 0.001). PICU admissions were higher in infants compared to the control group (*p* < 0.001). However, after adjusting for the type of surgery, no statistical difference was found. Additionally, there were no significant differences in the relative risk of ER visits, fever, or bleeding between the groups. A total of 60 patients (14.3%) in the cohort underwent reoperations, with higher rates in toddlers compared to infants and controls (25.2%, 16.4% and 6.2%, respectively, *p* < 0.001). The most common revision surgery for the control group was adenoidectomy (8/13, 61.5%), while for toddlers and infants, the most common revision surgery was adenoidectomy + tonsillectomy (22/37, 59.45% and 5/10, 50%, respectively). However, after adjusting for the type of surgery, no statistical difference was found.

**Conclusion:**

OSA surgery in children aged ≤ 2 years is generally safe but carries risks, including longer hospital stays. The higher rate of reoperations in this age group highlights the need for longer follow‐up and parental education about the recurrence of clinical symptoms.

## Key Points

- Infants underwent adenoidectomy alone more frequently, while older children had a higher rate of tonsillotomy with adenoidectomy.
- Infants (≤ 1 year) had significantly longer hospital stays (mean 3.9 days) and higher PICU admission rates (15%) compared to older children (0.9%).
- Lower postoperative complication rates in infants—Despite concerns about complications in younger children, no cases of postoperative bleeding or dehydration were recorded in infants, likely due to conservative surgical approaches and longer post‐op monitoring.
- Infants had a significantly higher reoperation rate (33%) compared to toddlers (23%) and older children (6.7%).
- The study found no significant difference in overall complication rates across age groups when adjusted for the type of surgery, suggesting that OSA surgery in children under 2 years is generally safe if appropriate surgical techniques and post‐op care measures are taken.

## 1 Background

While paediatric obstructive sleep apnea (OSA) is a widely studied disease, only a few studies have dealt with special sub‐groups of paediatric patients—infants (0–1 year) and toddlers (1–2 years). This distinct population usually represents different pathophysiology, co‐morbidities and treatment [1, 2].

One of the most common treatments for children diagnosed with OSA is surgery (adenoidectomy, tonsillectomy, or adenotonsillectomy) [3]. Complications following OSA surgeries have been widely studied and include postoperative haemorrhage, pain, dehydration and respiratory compromise [4, 5, 6, 7, 8]. There is a common belief that a higher risk for haemorrhage and respiratory complications exists for children under 2 years of age and weighing 14 kg [9, 10].

A recent Systematic Review and Meta‐analysis of the management of OSA in Infants revealed substantial heterogeneity in patient characteristics, diagnosis and interventions compared across multiple studies. Most studies included a small number of participants, making it hard to draw substantial conclusions [11].

Our study aimed to evaluate if surgery for OSA is safe for infants and toddlers as it is for older children.

## 2 Methodology

### 2.1 Ethical Considerations

This study was approved by the Institutional Ethical Review Board (0459‐23‐SOR). Due to the retrospective nature of the study, the ethics committee exempted the researchers from the need to obtain signed informed consent from the participants.

### 2.2 Study Design

This is a retrospective comparative analysis study at a single tertiary hospital aimed at comparing different outcomes for OSA surgery (tonsillectomy with adenoidectomy, tonsillectomy without adenoidectomy and adenoidectomy, ICD 9—28.2, 28.3, 28.6) among children in different age groups. Patients who needed surgery for laryngomalacia during the oSDB surgery were excluded from the study. Patients were divided into three age groups: study group 1—infants (age 0–12 months), study group 2—toddlers (1–2 years) and control group—children (over 2 years). Study groups 1 and 2 were composed of all patients ≤ 2 years of age who underwent OSA surgeries during 2014. The control group was patients > 2 years of age who underwent OSA surgeries during 2014 and were included as consecutive. The year 2014 was chosen in order to have a 10‐year follow‐up. We excluded patients with missing data, non‐OSA indication for surgery, or previous OSA surgeries. The indication for surgery was clinically assessed sleep‐disordered breathing (SDB) based on symptoms such as snoring, apnea, mouth breathing and failure to thrive (FTT), rather than a definitive diagnosis of OSA confirmed by polysomnography (PSG). PSG was not routinely used in our clinical setting for surgical decision‐making in healthy children under 2 years of age.

### 2.3 Postoperative Management

In our institution, postoperative admission plans are determined by patient age, comorbidities and the type of surgery, with occasional exceptions. As a general practice, any procedure that includes tonsillectomy—whether partial or total—requires at least one overnight stay in the paediatric surgical ward. Children under 2 years of age are not routinely treated as same‐day surgery cases; even when undergoing adenoidectomy alone, they are usually admitted for at least overnight observation.

Postoperative paediatric intensive care unit (PICU) admission may be planned preoperatively for high‐risk patients (e.g., very young age and significant comorbidities) as a precautionary measure. Alternatively, it may be decided postoperatively by the anaesthesiologist in the recovery room based on the child's clinical status (e.g., desaturations after extubation, hypotonia or other comorbidities, copious secretions in the airway, or unstable general condition).

### 2.4 Data Collection

Data extraction was performed using a standardised data collection form. The following variables were extracted:

– General demographics, medical background and clinical features: Age, sex, weight, underlying diseases (preterm birth, hypotonia, laryngomalacia, asthma, mucopolysaccharidosis), known coagulopathy, symptoms complex (snoring, apnea, rhinorrhea, mouth breathing, cough, FTT).
– Surgical details: Type of surgery (adenoidectomy with partial tonsillectomy, adenoidectomy with complete tonsillectomy, adenoidectomy without tonsillectomy, partial tonsillectomy without adenoidectomy, complete tonsillectomy without adenoidectomy).

Postoperative outcomes were: the need for PICU admission after surgery, and paediatric emergency department (ED) visits 14 days post‐surgery, with the following complications: bleeding, dehydration and fever. In addition, we examined if a reoperation was performed during the 10 years of follow‐up after the initial surgery.

### 2.5 Statistical Analysis

Data were analysed using descriptive statistics to summarise patient characteristics and complication rates within each age group. Continuous variables were presented as means, medians, standard deviations and ranges, while categorical variables were described in terms of frequencies and percentages. Continuous variables were compared using a *t*‐test for normally distributed data, and a Mann–Whitney test in cases where the distribution departs from normal. Categorical variables were compared using the Chi‐square test, with the use of the Fisher exact test when necessary. A *p* value of < 0.05 was considered statistically significant. Logistic regression analysis was then performed to adjust for potential confounders and to identify independent predictors of postoperative complications.

## 3 Results

A total of 419 patients met the inclusion criteria and were divided into three groups based on age: Infants, toddlers and control, with 61 (14.5%), 147 (35.1%) and 211 (50.4%) patients, respectively.

Demographics and medical background are presented in Table 1. Most patients were healthy males (67.3%), with no significant differences between the groups.

**TABLE 1:** Characteristics of study groups.

Characteristic	Control > 2 years, *N* = 211	Infants < 1 year, *N* = 61	*p* ^1^	Toddlers 1–2 years, *N* = 147	*p* ^2^
Gender, *n* (%)			0.3		0.2
Female	76 (36)	18 (30)		43 (29)	
Male	135 (64)	43 (70)		104 (71)	
Weight, kg			< 0.001		< 0.001
Mean ± SD	11.54 ± 1.78	8.33 ± 2.45		9.68 ± 1.67	
Median (IQR)	11.4 (10, 12.7)	8.05 (6.78, 9.63)		9.7 (8.84, 10.5)	
Range	7–18	4–16		4.5–15	
Background diseases, *n* (%)					
Healthy	167 (81)	45 (74)	0.4	109 (75)	0.3
Preterm (< 37 weeks)	7 (3.4)	6 (9.8)	0.08	17 (12)	0.002
Hypotonia	2 (1)	2 (3.3)	0.2	2 (1.4)	> 0.9
Laryngomalacia	—	5 (8.2)	< 0.001	2 (1.4)	0.2
Myasthenia gravis	—	1 (1.6)	0.2	—	—
Epilepsia	2 (1)	1 (1.6)	0.5	1 (0.7)	> 0.9
Nephropathy	5 (2.4)	1 (1.6)	> 0.9	1 (0.7)	0.4
Deafness	2 (1)	—	> 0.9	2 (1.4)	> 0.9
MPS	—	—	—	1 (0.7)	0.4
Asthma	6 (2.9)	—	0.3	6 (4.1)	0.6
Backwidth Weidman	—	—	—	1 (0.7)	0.4
Cerebral palsy	1 (0.5)	—	> 0.9	—	> 0.9
Pompe disease	—	—	—	1 (0.7)	0.4
Cardiopathy	7 (3.4)	—	0.4	2 (1.4)	0.3
Albinism	1 (0.5)	—	> 0.9	—	> 0.9
Hypospadias	1 (0.5)	—	> 0.9	—	> 0.9
Turner syndrome	1 (0.5)	—	> 0.9	—	> 0.9
VT shunt	1 (0.5)	—	> 0.9	—	> 0.9
Breath‐holding spells	1 (0.5)	—	> 0.9	—	> 0.9
Wolfarm syndrome	1 (0.5)	—	> 0.9	—	> 0.9
Down syndrome	1 (0.5)	—	> 0.9	—	> 0.9
Charge syndrome	1 (0.5)	—	> 0.9	—	> 0.9

*Note: p* ^1^ refers to the comparison between control (children > 2 years) and infants < 1 year. *p* ^2^ refers to the comparison between control (children > 2 years) and toddlers 1–2 years.

Infants presented a borderline significance compared with control in the prevalence of pre‐term birth (9.8% vs. 3.4%, *p* = 0.08, respectively), while toddlers demonstrated a significant difference compared with control (12% vs. 3.4%, *p* = 0.002, respectively). Laryngomalacia was significantly more common in infants compared to control (8.2% vs. 0%, *p* < 0.001, respectively).

**FIGURE 1:**
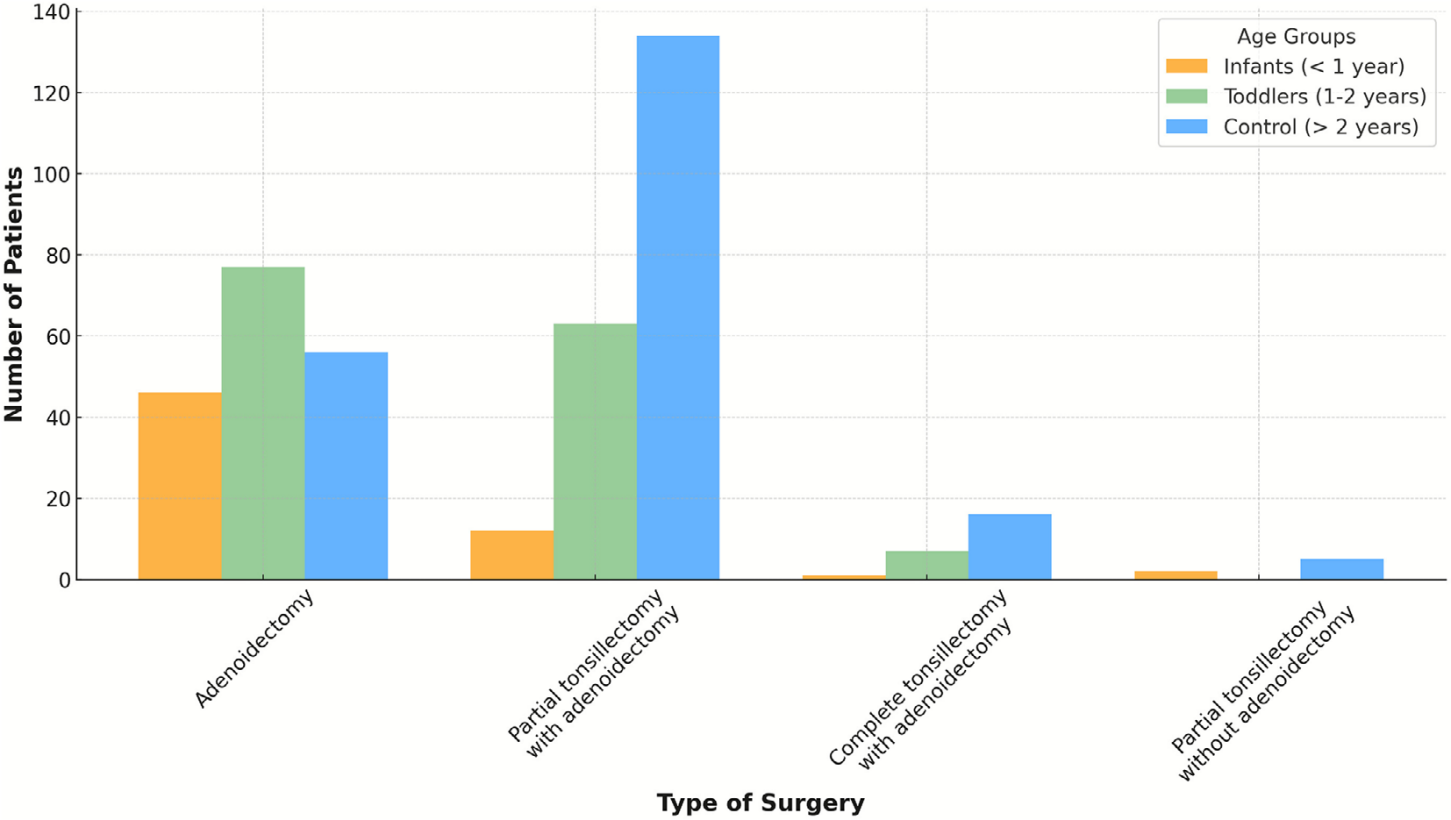
Type of OSA surgery.

There was a significant variation in the types of surgical procedures performed among the study groups (*p* < 0.001) (Figure 1). Infants mostly underwent adenoidectomy alone, toddlers most commonly underwent partial (intracapsular) tonsillectomy with adenoidectomy, and the control group mostly underwent tonsillectomy with adenoidectomy (134, 64%). In our cohort, the majority of tonsil surgeries were partial (intracapsular) rather than complete tonsillectomy.

Hospitalisation length of stay (LOS) was significantly higher in the infant group with a mean time of 3.9 (± 4.48) days, compared to toddlers 2.87 (± 0.91) days and control 2.09 (± 1.86) days (*p* < 0.001).

Surgical outcomes are presented in Table 2. Post‐operative PICU admissions were significantly higher in infants compared to controls (15% vs. 0.9%, *p* < 0.001, respectively). None of the patients admitted to the PICU suffered from respiratory or anaesthesia‐related complications. In our cohort, most PICU admissions were planned preoperatively as a precautionary measure when there were significant comorbidities, while some were decided postoperatively by the anaesthesiologist based on the child's condition.

**TABLE 2:** Surgical outcomes.

Characteristic	Control > 2 years, *N* = 211	Infants < 1 year, *N* = 61	*p* ^1^	Toddlers 1–2 years, *N* = 147	*p* ^2^
Length of stay, days			< 0.001		< 0.001
Mean ± SD	2.09 ± 1.86	3.9 ± 4.48		2.87 ± 0.91	
Median (IQR)	2 (1, 3)	3 (2, 3.25)		3 (2, 3)	
Range	1–25	1–35		2–8	
PICU, *n* (%)	2 (0.9)	9 (15)	< 0.001	4 (2.8)	0.2
Return to hospital, *n* (%)	17 (8.1)	2 (3.3)	0.3	18 (12)	0.2
Fever	9 (4.3)	2 (3.3)	> 0.9	11 (7.5)	0.2
Bleeding	7 (3.3)	—	0.4	7 (4.8)	0.5
Dehydration	1 (0.5)	—	> 0.9	1 (0.7)	> 0.9
Repeat surgery, *n* (%)	13 (6.2)	10 (16.4)	0.011	37 (25.2)	< 0.001
Adenoidectomy	8	4		13	
Tonsillectomy	1	1		2	
Adenoidectomy + Tonsillectomy	4	5		22	

*Note: p* ^1^ refers to the comparison between control (children > 2 years) and infants < 1 year. *p* ^2^ refers to the comparison between control (children > 2 years) and toddlers 1–2 years. PICU—hospitalisation in the paediatric intensive care unit after the surgery.

Overall, the rate of ED visits up to 14 days following surgery was 8.8% (37/419). Bleeding concerns were the cause of the ED visit in 37.8% (14/37) of patients (3.3% of all the patients in the study). In the infant group, none of the patients suffered from bleeding or dehydration after surgery.

Regarding late outcomes, 60 (14.3%) patients underwent reoperation during the 10‐year follow‐up period. Higher reoperation rates were recorded in the infant group (10, 16.4%) compared to toddlers and control (37, 25% and 13, 6.2%, respectively) *p* < 0.001. The most common revision surgery for the control group was adenoidectomy (8/13, 61.5%), while for toddlers and infants, the most common revision surgery was adenoidectomy + tonsillectomy (22/37, 59.45% and 5/10, 50%, respectively).

Table 3 summarises the relative risk (RR) of adverse postoperative Outcomes, adjusted for the type of surgery. LOS was still statistically significant both for the infant group (*RR* = 1.96) and for the toddler group (*RR* = 1.44) compared to control (*p* < 0.001).

**TABLE 3:** Postoperative outcomes in infants and toddlers compared to control, adjusted for type of surgery.

Medical condition	Infants < 1 year			Toddlers 1–2 years		
	RR for age group	95% CI	*p* ^1^	RR for age group	95% CI	*p* ^2^
Length of stay	1.96	1.64–2.35	< 0.001	1.44	1.25–1.65	< 0.001
PICU	1.14	0.84–1.53	0.4	1.02	0.82–1.27	0.9
Return to hospital	0.55	0.08–2.17	0.4	1.68	0.84–3.4	0.14
Fever	0.9	0.12–4.2	> 0.9	1.71	0.67–4.56	0.3
Bleeding	—	—	—	1.73	0.59–5.07	0.3
Hypohydration	—	—	—	2.13	0.13–34	0.6
Repeat surgery	1.22	0.91–1.62	0.2	1.13	0.92–1.38	0.2

*Note: p* ^1^ refers to the comparison between control (children > 2 years) and infants < 1 year. *p* ^2^ refers to the comparison between control (children > 2 years) and toddlers 1–2 years. PICU—hospitalisation in the paediatric intensive care unit after the surgery.

As for admission to PICU and reoperation, which were statistically different in Table 2, when adjusted for the type of surgery, no statistical difference was seen.

## 4 Discussion

Complications following OSA surgeries are a concern for clinicians and parents. For younger children, this has become a major concern and has led to restricted guidelines that sometimes can postpone the treatment for OSA [12]. During the last few decades, surgical techniques have improved dramatically, and thus the complication rates have declined [13]. In this study, we evaluated the different outcomes and complications of OSA surgery according to stratified age groups.

Our main findings include a higher rate of LOS for infants and toddlers, higher rates of PICU admission for infants and significantly higher rates of reoperation during the 10‐year follow‐up period for infants, reaching up to 33%. No difference was found regarding all other complications, suggesting an overall safety for OSA surgeries even among the youngest age group.

An interesting finding that differed between the groups (although not statistically significant) was higher co‐morbidities and more preterm births in infants compared to children. It is known that some comorbidities are associated with OSA in infants [1]. This result is not surprising, as prematurity is associated with reduced muscle tone and a higher prevalence of upper airway symptoms in infancy, which may contribute to earlier presentation and surgical referral. The authors assume that children with comorbidities present at a younger age and with more symptoms, such as FTT, loud snoring at night and even apnea that the parents can notice. Another possible explanation for this difference is that in more ‘healthy’ children, surgery may be postponed in order to achieve a PSG as the guidelines recommend [12], causing a delay in treatment and an older age at the time of surgery [14]. This study demonstrates significant differences between the groups in the type of surgery. Infants had more adenoidectomy alone, toddlers had almost equal percentages between adenoidectomy and adenoidectomy with partial tonsillectomy, and in children, the majority had adenoidectomy with partial tonsillectomy. This has several possible explanations. The first is anatomical. At a younger age, the tonsils still did not grow enough to play a major role in the pathogenesis of OSA; thus, there was no need to address them in the surgery [15, 16]. The second possible explanation is concern about complications, especially bleeding [10]. This can influence this study's outcomes, both in complication rate and in the need for reoperation, as will be addressed later in the discussion.

An inverse relationship was observed between age group and LOS, with infants exhibiting the longest stays, toddlers having intermediate stays and children having the shortest. This may be attributed to concerns regarding dehydration, as highlighted in previous studies [1, 2, 5, 9, 10, 11], and the necessity to ensure that infants consume adequate food and fluids prior to hospital discharge. It can be inferred that this precautionary approach contributed to a lower complication rate in the younger age groups.

This study found significantly higher PICU admissions in infants (9, 15%) compared to children (2, 0.9%) (*p* < 0.001). This difference did not appear when adjusted for the type of surgery. Although we found this difference, none of the children in our cohort developed postoperative respiratory complications or anaesthesia‐related complications. A systematic review and meta‐analysis published by Keserű et al. examined the risk of postoperative respiratory complications following adenotonsillar surgery in children with OSA [17]. They concluded that children with moderate and severe OSA are more likely to develop postoperative respiratory complications following surgery, but no significant difference was found in mild OSA. In 2016, however, the FDA issued a warning on the use of general anaesthesia in young children [18]. The warning is specific to repeated or lengthy use of general anaesthetic and sedation drugs during surgeries or procedures in children less than 3 years old. In a paper published in 2021 about the safety of anaesthesia in young children presenting for adenotonsillectomy [19], they stated that many things can be done to mitigate these risks, such as optimising the patient pre‐operatively, as well as recognising patients at an increased risk for perioperative complications secondary to OSA or other comorbidities.

Concerning other surgical outcomes, postoperative admission to the ED 2 weeks after surgery did not differ significantly between the groups. When we examined the type of complications (bleeding, fever, dehydration), we still did not find any significant difference. Our overall complication rates correspond to the numbers previously reported in the literature but differ from other studies, which found a higher risk for complications in younger patients [10]. Interesting to note that in the infant group, no bleeding or dehydration was seen. We assume that the difference in our results compared to other cohorts [10, 20] lies in a less aggressive surgical treatment: more partial tonsillectomy versus complete tonsillectomy, and more adenoidectomy alone versus adenoidectomy combined with tonsillectomy, which are known to cause fewer complications post‐surgery [21, 22, 23, 24]. It can also be an anatomical difference between the groups, as younger patients had much fewer upper respiratory episodes and less neogenesis of blood vessels in the surgical site, which can lead to post‐surgical bleeding [15, 16]. When examining the RR of adverse postoperative medical conditions, adjusted for the type of surgery, we did not find a statistically significant difference. Our findings suggest that it is a safe method to treat OSA under 2 years of age, and even under 1 year of age if the proper measures are taken: partial tonsillectomy rather than total, longer hospitalisation after surgery and educating the parents on all aspects of post‐surgery care.

As for reoperation, 14.3% of our cohort underwent reoperation during the follow‐up period. Higher rates were seen in toddlers (25.2%) compared to infants (16.4%) and the control group (6.2%). This was anticipated and probably related directly to our initial surgical method, as we did not see this difference when comparing the rates adjusted for the type of surgery. Patients who had only adenoidectomy (most of the infant group) were at a greater risk for tonsillar hypertrophy later in their lives, compared to patients who had partial or total tonsillectomy (most of the control group). It is important to explain to parents that there could be a need for surgery a few years after the initial one, and to follow the recurrence of clinical manifestations.

### 4.1 Limitations

The major limitation of our study is the number of patients, although relatively large. Since the complication rates are minimal, a larger database is needed to support our findings and to allow for a change in the medical guidelines. Our study was conducted at a regional tertiary care centre with access to a dedicated paediatric surgical ward and a PICU. As such, the findings may not be fully generalizable to smaller centres or community hospitals that lack such resources. The safety outcomes observed in our cohort may reflect, in part, the availability of specialised perioperative monitoring and paediatric critical care. Additional limitation derives from its retrospective nature and can lead to bias in the patient selection, treatment selection and the availability of the data. Minor postoperative bleeding events that resolved spontaneously during the initial hospitalisation and did not require physician assessment were not routinely documented in the medical records and, therefore, were not captured in our analysis. Lastly, another limitation is that the diagnosis of OSA was not based on objective PSG findings, but rather on clinical symptoms consistent with SDB. While this approach reflects real‐world surgical decision‐making in many paediatric centres, it limits our ability to stratify outcomes by OSA severity or to generalise our findings to children with PSG‐confirmed OSA.

## 5 Conclusion

OSA surgery for children ≤ 2 years of age is a safe method, but not without risks, especially longer LOS. Higher rates of reoperations in this age group necessitate a longer follow‐up period and parental education regarding the recurrence of clinical manifestations. Therefore, it is crucial to weigh the patient's individual risks versus the benefits of the surgery. A prospective large‐scale study is needed to further explore the subject.

## Author Contributions

Daniel Levi – data collection, Helsinki approval, drafting the article. Daniel Yafit – study design, drafting and revisions of the article. Aviad Sapir – data collection, drafting the article. Yotam Heilig – data collection, drafting the article. Tomer Kerman – statistical analysis. Oriya Damri – data collection. Inbal Golan‐Tripto – study design, drafting and revisions of the article. Daniel Michael Kaplan – study design, drafting and revisions of the article. Oren Ziv – study design, drafting and revisions of the article.

## Funding

The authors have nothing to report.

## Ethics Statement

The study was approved by the SOROKA Medical Center institutional Helsinki committee—0459‐23‐SOR.

## Consent

The institutional Helsinki committee approved the lack of need for consent to participate due to the retrospective nature of the study. All authors have made consent to publish the study.

## Conflicts of Interest

The authors declare no conflicts of interest.

## Data Availability

The data that support the findings of this study are available on request from the corresponding author. The data are not publicly available due to privacy or ethical restrictions.
